# Development and internal-external validation of a risk prediction nomogram for secondary myocardial injury in traumatic brain injury

**DOI:** 10.3389/fneur.2026.1770629

**Published:** 2026-03-12

**Authors:** Yu-Qin Zhan, Chen-yang Wu, Yu-bin Shen, Ya-hui Ding

**Affiliations:** 1 The 2nd Clinical Medical College of Zhejiang Chinese Medical University, Hangzhou, Zhejiang, China; 2 Department of Cardiovascular Medicine, Heart Center, Zhejiang Provincial People’s Hospital (Affiliated People’s Hospital, Hangzhou Medical College), Hangzhou, Zhejiang, China

**Keywords:** intensive care unit, secondary myocardial injury, nomogram, predictive model, traumatic brain injury

## Abstract

**Introduction:**

Traumatic Brain Injury (TBI)-induced secondary myocardial injury (SMI) is a severe complication with poor prognosis, but reliable early predictive tools are lacking. This study aimed to develop and validate a nomogram for predicting this risk in TBI patients admitted to the intensive care unit (ICU).

**Methods:**

We retrospectively analyzed 1,042 ICU-admitted TBI patients without pre-existing cardiac disease from the MIMIC-IV database, randomly divided into training (*n* = 729) and internal validation (*n* = 313) sets at a 7:3 ratio. An external validation cohort of 200 patients from Zhejiang Provincial People’s Hospital (2020–2025) was also included. Five key predictors were identified via univariate and multivariate logistic regression.

**Results:**

The final model included blood urea nitrogen, hemoglobin, Sequential Organ Failure Assessment (SOFA) score, serum potassium, and creatinine. It showed good discriminative ability: training set AUC = 0.772 (95%CI: 0.737–0.808), internal validation set AUC = 0.785 (95%CI: 0.733–0.837), and external validation set AUC = 0.848 (95%CI: 0.778–0.917).

**Conclusions and discussion:**

This nomogram, based on easily accessible clinical parameters, enables early risk stratification of SMI in TBI patients before cardiac biomarker elevation, providing a practical tool for targeted clinical monitoring and intervention.

## Introduction

Traumatic brain injury (TBI) is a major global public health issue and ranks among the leading causes of mortality and disability globally. Defined as an alteration in brain function or neuropathological changes resulting from external forces1. TBI affects approximately 69 million people annually worldwide. Incidence varies substantially across European countries (47–850 per 100,000 population), while China reports over 3–4 million cases each year (1).

TBI-induced myocardial injury differs from primary cardiac disease due to its unique pathophysiological basis: abnormal activation of the “brain-heart axis” rather than traditional factors like coronary artery disease. Following TBI, primary neural injury triggers systemic inflammatory responses, autonomic dysfunction, and metabolic disorders, which mediate myocardial injury through two key pathways. On one hand, overactivation of the hypothalamic–pituitary–adrenal axis and sympathetic hyperactivity induce a “catecholamine storm,” directly causing myocardial cell necrosis, calcium overload, and mitochondrial dysfunction (2); on the other hand, damage—associated molecular patterns (DAMPs) released from brain tissue injury activate immune cells to release inflammatory factors such as tumor necrosis factor-α (TNF-α) and interleukin-6 (IL-6), which exacerbate myocardial inflammation and apoptosis through the circulatory system (3).

This unique mechanism leads to TBI severity-dependent incidence of myocardial injury: meta-analyses show an overall incidence of 33% (95%CI:27–39%) in adult TBI patients with 19% in mild cases, 29% in moderate-to-severe cases, and up to 54% in severe cases (4). Elevated cardiac troponin (cTn)—a biomarker of this injury—is a key indicator of poor prognosis, correlating with increased mortality risk even in mild TBI patients (5, 6). TBI patients who develop myocardial injury during intensive care unit (ICU) stay face prolonged hospitalization and significantly higher mortality, underscoring the need for early identification and intervention (7).

Despite extensive research on the risks and prognosis of post-TBI myocardial injury (8–10), and the identification of independent risk factors for cardiogenic shock (CCS) (11). Existing ICU myocardial injury prediction models are mostly designed for general critically ill patients, failing to account for TBI-specific “brain-heart axis” dysregulation and common comorbidities such as electrolyte disturbances (e.g., hyperkalemia) and renal function changes (12). Additionally, traditional cTn assays have reduced sensitivity and specificity in TBI, creating a “diagnostic gap” that hinders early risk stratification (11).

Therefore, this study aims to develop and validate a nomogram specifically designed to predict the risk of SMI in adult traumatic brain injury (TBI) patients admitted to the ICU, using readily available clinical and laboratory parameters from the MIMIC-IV database. Its core innovations are as follows: (1) Based on the pathophysiological mechanism of myocardial injury mediated by the unique “brain-heart axis” in TBI patients; (2) Utilizing accessible clinical and laboratory indicators (e.g., the SOFA score reflecting systemic inflammation, and electrolyte parameters associated with brain-heart axis regulation) as predictors, rather than relying on delayed biomarkers or complex imaging studies; (3) Selecting key variables via univariate and multivariate logistic regression to construct and validate the nomogram for predicting SMI in adult TBI patients during ICU hospitalization.

## Materials and methods

### Data source

Data for this study were extracted from the MIMIC-IV v2.2 database^1^ (13), which contains comprehensive health records of more than 50,000 patients admitted to ICUs at Beth Israel Deaconess Medical Center between 2008 and 2019. We specifically selected patients diagnosed with TBI who were admitted to the ICU and identified using ICD-10 codes S06 and ICD-9 code 85. This coding practice aligns with the structure of the database and previous studies utilizing the MIMIC-IV for TBI research (14). The database includes vital signs, laboratory parameters, medication regimens, diagnostic codes, imaging reports, length of hospitalization, mortality data, and other clinically relevant information. Patient consent was not required for this study as the use of this de-identified database was approved by the Institutional Review Boards of both the Massachusetts Institute of Technology and Beth Israel Deaconess Medical Center. Upon successful completion of the necessary evaluation (Certificate No. 32173796), the individuals involved in this study were granted authorization to access the MIMIC-IV database after completing a series of courses provided by the National Institutes of Health (NIH).

### Study populations

The study population consisted of patients admitted to the intensive care unit (ICU) with confirmed traumatic brain injury (TBI). Patients with or without concomitant extracranial injuries were eligible for inclusion if, they met the criteria. The presence and severity of extracranial injuries were not exclusion criteria for this study, as the primary focus was on myocardial injury in patients with TBI within the ICU setting, a population that frequently presents with polytrauma. The inclusion criteria were as follows:

1. Patients identified with TBI using International Classification of Diseases (ICD) diagnostic codes.
2. Patients experiencing their first ICU admission with a minimum length of stay of 3 h.
3. Adult patients aged 18 years or older.
4. Patients who underwent troponin testing following ICU admission.
5. Patients without a history of underlying cardiac conditions, specifically chronic ischemic heart disease, previous myocardial infarction, end-stage renal disease (ESRD) or dialysis dependence, or severe sepsis.

A total of 1,042 TBI patients were included in the analysis. The cohort was randomly divided into training and validation cohorts in a 7:3 ratio, with the training cohort used to develop the nomogram and the validation cohort used for validation purposes. Additionally, to evaluate the generalizability of the model, an external validation cohort comprising 200 adult patients with TBI admitted to Zhejiang Provincial People’s Hospital between January 2020 and January 2025 was retrospectively enrolled. These patients were selected using the same inclusion and exclusion criteria as those used for the MIMIC-IV cohort, including the requirement for first ICU admission, age ≥18, and absence of pre-existing cardiac conditions.

### Data extraction

We utilized the Structured Query Language (SQL) in Navicat Premium version 15 to extract necessary data from the MIMIC-IV database. All extracted data underwent rigorous validation and filtering in accordance with best practices for scientific computation. The extracted information included as following:

1. Demographics: age and sex, which are essential for analyzing disease distribution and outcomes across different populations and providing a foundation for assessing disease impact;
2. Comorbidities: diabetes, kidney disease, and pulmonary disease;
3. Laboratory tests: white blood cell count, red blood cell count, hemoglobin, platelets, creatinine, glucose, sodium, calcium, potassium, anion gap, and coagulation function parameters, which collectively reflect patients’ physiological and metabolic status;
4. Vital signs: systolic blood pressure, diastolic blood pressure, heart rate, respiratory rate, and body temperature;
5. Disease severity assessments: Glasgow Coma Scale (GCS), Sequential Organ Failure Assessment (SOFA), and intracranial pressure (ICP), which are validated scoring systems used to evaluate disease severity upon ICU admission (15–18).

Predictor Measurement and Timing Time zero was defined as the time of ICU admission. To ensure the model’s applicability for early risk stratification, all predictor variables were extracted from data recorded within the first 24 h of ICU admission. For continuous variables with multiple measurements during this window, the “worst” value was selected to represent the patient’s acute severity.

#### Missing data management

Missing data were prevalent in the MIMIC-IV database. We summarized the missing rates for all candidate variables in Supplementary Table S1. For continuous variables, we employed different imputation strategies based on the extent of missingness: mean imputation for variables with <5% missing values, multiple imputation using the R package “mice” (with 5 iterations) for variables with 5–25% missing data, and complete exclusion for variables with >25% missing values (such as intracranial pressure, pH, and C-reactive protein) to minimize potential bias. For categorical variables, missing values were categorized as “Unknown” to preserve information integrity. Outliers were considered as missing values and processed using the aforementioned strategies.

### Definition of secondary myocardial injury

In this study, the outcome was operationally defined as “Secondary Myocardial Injury” (SMI), aligning with the criteria for acute myocardial injury outlined in the Fourth Universal Definition of Myocardial Infarction (19–21), characterized by marker elevation without requiring concurrent evidence of myocardial ischemia. This broad definition captures the heterogeneous etiologies of cardiac damage in TBI, including neurogenic stunning and catecholamine toxicity. SMI was identified by cardiac troponin (cTn) levels exceeding the 99th percentile upper reference limit (URL) during ICU admission. Adopted cutoffs based on MIMIC-IV standards were: hs-TnT > 14 ng/L (men) and > 34 ng/L (women); or cTnI > 0.04 ng/mL. Given the retrospective nature of the study and the inability to reliably assess ischemic symptoms in comatose patients, this objective laboratory-based definition minimizes selection bias compared to definitions requiring imaging (echocardiography) or symptom reporting.

### Data analysis

The samples were randomly allocated to training and validation sets in a 7:3 ratio using the “caret” package in R software to perform stratified random sampling, ensuring that the distribution of SMI events remained consistent across both subsets. Variables were characterized separately in both datasets. We performed a stratified analysis to compare the SMI and non-SMI groups. Categorical variables were presented as percentages (%), non-normally distributed continuous variables as medians with interquartile ranges, and normally distributed continuous variables as means with standard deviations. Differences between categorical variables were assessed using chi-square tests, while differences in continuous variables between groups were evaluated using *t*-tests or nonparametric tests as appropriate. Univariate logistic regression models were constructed using the training set, and variables with *p* values < 0.05 and variance inflation factors (VIF) < 5 were included in the multivariate logistic regression model. Subsequently, multivariate logistic regression was performed using a stepwise forward selection method. Based on this, a secondary screening was performed considering clinical practicality and model parsimony to finalize the predictive variables included in the model. The area under the receiver operating characteristic curve (AUC) was calculated to assess the model’s predictive accuracy. Calibration curves were employed to evaluate consistency between predicted and actual values, while decision curve analysis (DCA) was conducted to assess the clinical utility of both the model and the nomogram. To further quantify the contribution of each predictor to the model’s output and clarify the relationships between variables and the risk of Secondary Myocardial Injury (SMI), we employed SHapley Additive exPlanations (SHAP) analysis to validate the interpretability of the final logistic regression model. Two-tailed *p* values < 0.05 were considered statistically significant. All statistical analyses were performed using SPSS software (version 27, IBM) and R software (version 4.4.1), utilizing packages including “compareGroups,” “caret,” “rms,” “mice,” “pROC,” “regplot,” “calibration,” “ResourceSelection,” “rmda,” “shap,” and “ggplot2.”

## Results

### Cohort characteristics

**Figure 1:**
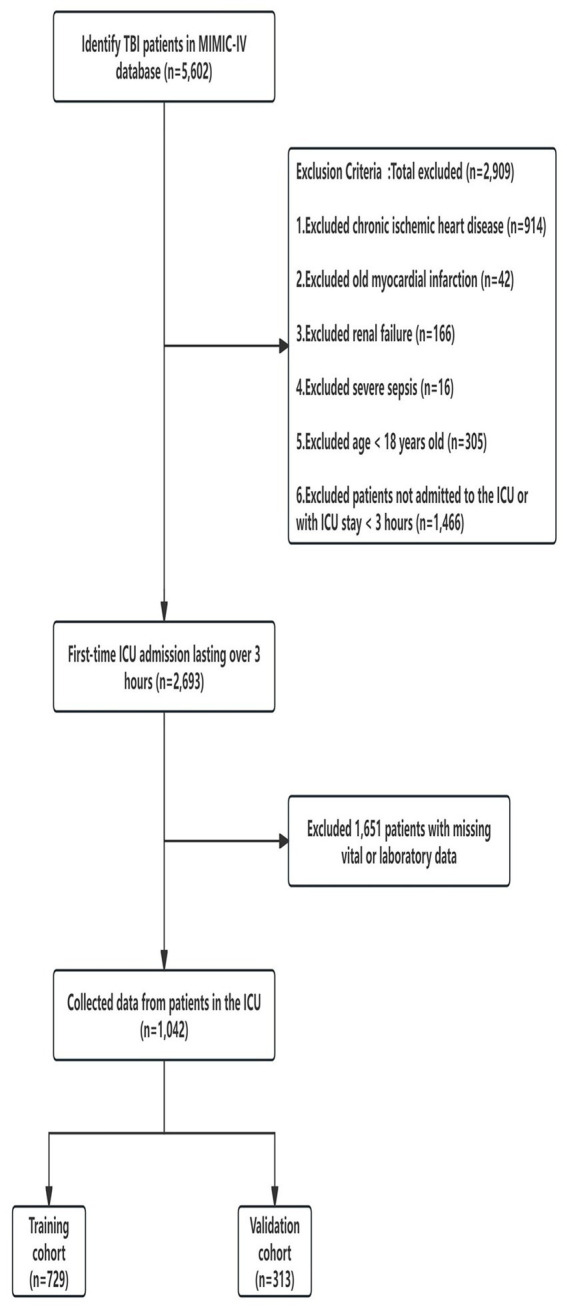
Flowchart of the study.

A total of 5,602 patients with TBI were initially identified in the MIMIC-IV database. Following the application of the inclusion and exclusion criteria, including the removal of cases with missing data and patients not admitted to the ICU, 1,042 patients were ultimately enrolled in the study. The study population was divided into a training cohort (*n* = 729, 285 patients with SMI) and a validation cohort (*n* = 313, 128 patients with SMI) (Figure 1). The baseline characteristics of both cohorts are summarized in Table 1. Comparative analysis revealed no statistically significant differences in the baseline characteristics between the training and validation cohorts (all *p* > 0.05).

**Table 1:** Baseline characteristics of training set and validation set.

	Overall	Training set	Validation set	*P*
*N*	1,042	729	313	
Gender (%)				0.973
Female	402 (38.58)	281 (38.55)	121 (38.66)	
Male	640 (61.42)	448 (61.45)	192 (61.34)	
Age (median [IQR])	74.00 [61.00, 84.00]	75.00 [62.00, 84.00]	72.00 [59.00, 83.00]	0.381
Invasive ventilation = Yes (%)	413 (39.64)	285 (39.09)	128 (40.89)	0.566
Gcs (%)				0.892
13–15	708 (67.95)	496 (68.04)	212 (67.73)	
9–12	202 (19.39)	139 (19.07)	63 (20.13)	
3–8	132 (12.67)	94 (12.89)	38 (12.14)	
Diabetes = Yes (%)	341 (32.73)	230 (31.55)	111 (35.46)	0.217
Chronic kidney disease = Yes (%)	262 (25.14)	182 (24.97)	80 (25.56)	0.840
Chronic pulmonary disease = Yes (%)	221 (21.21)	149 (20.44)	72 (23.00)	0.353
SOFA (median [IQR])	3.00 [2.00, 5.00]	3.00 [2.00, 5.00]	3.00 [2.00, 5.00]	0.616
DBP (mmHg) (median [IQR])	66.33 [58.25, 74.46]	65.98 [58.44, 74.12]	66.81 [58.04, 75.69]	0.515
SBP (mmHg) (median [IQR])	127.58 [116.89, 138.07]	127.20 [117.64, 138.14]	128.27 [115.96, 137.56]	0.818
Heart Rate (median [IQR])	83.69 [73.94, 95.68]	84.00 [73.23, 96.00]	83.41 [74.61, 9,552]	0.697
Resp rate (median [IQR])	18.96 [17.11, 21.28]	18.87 [17.00, 21.20]	19.15 [17.19, 21.81]	0.189
Temperature (°C) (median [IQR])	36.98 [36.70, 37.29]	36.97 [36.70, 37.29]	36.99 [36.69, 37.27]	0.947
Height (cm) (median [IQR])	170.00 [163.00, 178.00]	170.00 [163.00, 178.00]	170.00 [163.00, 178.00]	0.655
Weight (kg) (median [IQR])	75.00 [63.23, 88.00]	74.80 [63.40, 88.10]	75.40 [63.00, 88.00]	0.833
BMI (kg/m^2^) (median [IQR])	27.05 [23.89, 30.76]	27.05 [23.85, 30.76]	27.08 [23.94, 30.36]	0.893
Hemoglobin (g/dL) (median [IQR])	9.80 [7.93, 11.30]	9.80 [8.00, 11.30]	9.70 [7.80, 11.30]	0.489
WBC (K/μL) (median [IQR])	11.70 [8.60, 16.00]	11.70 [8.50, 15.80]	12.00 [8.70, 16.00]	0.583
Platelet (K/μL) (median [IQR])	159.00 [119.00, 206.00]	161.00 [120.00, 207.00]	152.00 [117.00, 200.00]	0.158
RBC (m/μL)(median [IQR])	3.20 [2.62, 3.73]	3.19 [2.61, 3.72]	3.22 [2.64, 3.77]	0.881
Lymphocytes (%) (median [IQR])	1.00 [0.67, 1.45]	1.01 [0.70, 1.45]	0.99 [0.60, 1.44]	0.255
Bicarbonate (mEq/L) (median [IQR])	25.00 [23.00, 27.00]	25.00 [23.00, 27.00]	25.00 [23.00, 27.00]	0.985
Creatinine (mg/dL) (median [IQR])	1.10 [0.80, 1.50]	1.10 [0.80, 1.50]	1.10 [0.80, 1.50]	0.846
Aniongap (mEq/L) (median [IQR])	17.00 [15.00, 20.00]	17.00 [14.00, 19.00]	17.00 [15.00, 20.00]	0.249
BUN (mg/dL) (median [IQR])	21.00 [16.00, 32.00]	21.00 [15.00, 32.00]	22.00 [16.00, 32.00]	0.292
Calcium(mg/dL) (median [IQR])	8.90 [8.40, 9.30]	8.90 [8.40, 9.30]	8.90 [8.40, 9.20]	0.758
Glucose (mg/dL) (median [IQR])	152.50 [125.00, 191.00]	154.00 [125.00, 192.00]	151.00 [125.00, 188.00]	0.626
Potassium (mEq/L) (median [IQR])	4.50 [4.10, 5.00]	4.50 [4.10, 5.00]	4.40 [4.10, 5.00]	0.885
Sodium (mEq/L) (median [IQR])	141.00 [139.00, 144.00]	141.00 [139.00, 144.00]	141.00 [139.00, 145.00]	0.428
PT (s) (median [IQR])	13.50 [12.30, 15.90]	13.50 [12.30, 15.60]	13.60 [12.20, 16.80]	0.533
PTT (s) (median [IQR])	29.60 [26.80, 34.20]	29.60 [26.80, 34.10]	29.50 [26.70, 35.10]	0.968
AST (IU/L) (median [IQR])	31.00 [31.00, 43.00]	31.00 [31.00, 43.00]	31.00 [31.00, 39.00]	0.582
ALT (IU/L) (median [IQR])	28.00 [28.00, 28.00]	28.00 [28.00, 28.00]	28.00 [27.00, 28.00]	0.913

IQR, interquartile range; GCS, Glasgow coma scale; SBP, systolic blood pressure; DBP, diastolic blood pressure; SOFA, sequential organ failure assessment; WBC, White blood cell; BUN, blood urea nitrogen; PT, prothrombin time; PTT, activated partial thromboplastin time; ALT, alanine aminotransferase; AST, aspartate aminotransferase.

### Comparison of baseline characteristics and outcomes between SMI and non-SMI groups

Among 1,042 TBI patients enrolled in this study, 413 (39.63%) developed secondary myocardial injury (SMI), while 629 (60.37%) did not (non-SMI group). Comparison of baseline characteristics between the two groups is presented in Table 2. Compared with the non-SMI group, SMI patients exhibited greater systemic inflammation (higher white blood cell count, blood glucose, potassium, and anion gap; lower lymphocyte count), worse organ function (elevated serum creatinine, BUN, AST, and ALT; prolonged PT and PTT; reduced platelet count), and more severe comorbidity burden (older age; higher prevalence of chronic kidney disease, chronic lung disease, congestive heart failure, and diabetes; higher GCS 3–8 proportion and SOFA scores) (all *p* < 0.05). They also showed compromised hematologic and circulatory status (lower hemoglobin and red blood cell counts; higher heart rate and respiratory rate; lower diastolic blood pressure) (all *p* < 0.05). Regarding outcomes, SMI was associated with significantly higher 30-day mortality (16.46% vs. 6.68%, *p* < 0.001) and longer hospital stay (7.71 vs. 6.80 days, *p* = 0.021).

**Table 2:** Baseline characteristics and outcomes of SMI versus non-SMI.

Variables	Total (*n* = 1,042)	Non-SMI (*n* = 629)	SMI (*n* = 413)	*P*
Hemoglobin, (g/dL) M (Q₁, Q₃)	9.80 (7.93, 11.30)	10.20 (8.70, 11.60)	8.70 (7.30, 10.40)	<0.001
WBC, (K/μL) M (Q₁, Q₃)	11.70 (8.60, 16.00)	11.40 (8.40, 15.20)	12.30 (8.80, 18.00)	0.002
Platelet, (K/μL) M (Q₁, Q₃)	159.00 (119.00, 206.00)	167.00 (128.00, 213.00)	150.00 (103.00, 188.00)	<0.001
RBC, (m/μL) M (Q₁, Q₃)	3.20 (2.62, 3.73)	3.37 (2.79, 3.83)	2.92 (2.42, 3.45)	<0.001
SOFA, M (Q₁, Q₃)	3.00 (2.00, 5.00)	3.00 (2.00, 4.00)	4.00 (2.00, 6.00)	<0.001
DBP, (mmHg) M (Q₁, Q₃)	66.33 (58.25, 74.46)	67.18 (58.97, 75.89)	64.88 (57.42, 73.50)	0.001
SBP, (mmHg) M (Q₁, Q₃)	127.58 (116.89, 138.07)	129.36 (117.72, 138.12)	125.80 (115.79, 137.26)	0.050
Heart Rate, M (Q₁, Q₃)	83.69 (73.94, 95.68)	82.00 (72.53, 94.91)	86.51 (75.54, 98.32)	<0.001
Resp Rate, M (Q₁, Q₃)	18.96 (17.11, 21.28)	18.54 (16.83, 20.57)	19.72 (17.38, 22.67)	<0.001
Temperature, (°C) M (Q₁, Q₃)	36.98 (36.70, 37.29)	37.01 (36.74, 37.29)	36.92 (36.66, 37.29)	0.022
Height, (cm) M (Q₁, Q₃)	170.00 (163.00, 178.00)	170.00 (163.00, 178.00)	170.00 (163.00, 178.00)	0.274
Weight, (kg) M (Q₁, Q₃)	75.00 (63.23, 88.00)	76.00 (64.10, 89.70)	72.90 (62.10, 86.80)	0.036
BMI, (kg/m^2^) M (Q₁, Q₃)	27.05 (23.89, 30.76)	27.08 (23.94, 30.84)	27.01 (23.80, 30.35)	0.610
Age, M (Q₁, Q₃)	74.00 (61.00, 84.00)	71.00 (58.00, 82.00)	78.00 (65.00, 86.00)	<0.001
Lymphocytes Min, (%) M (Q₁, Q₃)	1.00 (0.67, 1.45)	1.01 (0.73, 1.51)	0.91 (0.61, 1.37)	0.001
Bicarbonate Max, (mEq/L) M (Q₁, Q₃)	25.00 (23.00, 27.00)	25.00 (23.00, 27.00)	25.00 (23.00, 28.00)	0.211
Creatinine Max, (mg/dL) M (Q₁, Q₃)	1.10 (0.80, 1.50)	1.00 (0.80, 1.20)	1.40 (1.00, 2.20)	<0.001
Aniongap Max, (mg/dL) M (Q₁, Q₃)	17.00 (15.00, 20.00)	16.00 (14.00, 19.00)	18.00 (15.00, 20.00)	<0.001
BUN Max, (mg/dL) M (Q₁, Q₃)	21.00 (16.00, 32.00)	18.00 (14.00, 25.00)	30.00 (20.00, 49.00)	<0.001
Calcium, (mg/dL) M (Q₁, Q₃)	8.90 (8.40, 9.30)	8.90 (8.40, 9.30)	8.80 (8.40, 9.30)	0.898
Glucose Max, (mg/dL) M (Q₁, Q₃)	152.50 (125.00, 191.00)	147.00 (120.00, 178.00)	166.00 (135.00, 221.00)	<0.001
Potassium Max, (mEq/L) M (Q₁, Q₃)	4.50 (4.10, 5.00)	4.40 (4.00, 4.80)	4.70 (4.20, 5.40)	<0.001
Sodium Max, (mEq/L) M (Q₁, Q₃)	141.00 (139.00, 144.00)	141.00 (139.00, 144.00)	141.00 (139.00, 144.00)	0.359
PT Max, (s) M (Q₁, Q₃)	13.50 (12.30, 15.90)	13.10 (12.10, 14.80)	14.20 (13.00, 18.50)	<0.001
PTT Max, (s) M (Q₁, Q₃)	29.60 (26.80, 34.20)	28.80 (26.40, 32.60)	31.30 (27.60, 36.80)	<0.001
AST, (IU/L) M (Q₁, Q₃)	43.00 (28.00, 83.00)	39.00 (26.25, 71.00)	48.00 (29.00, 109.00)	0.001
ALT, (IU/L) M (Q₁, Q₃)	28.00 (17.00, 48.00)	26.00 (16.00, 43.00)	32.00 (18.00, 61.00)	0.006
Gender, *n* (%)				0.664
Female	402 (38.58)	246 (39.11)	156 (37.77)	
Male	640 (61.42)	383 (60.89)	257 (62.23)	
Diabetes, *n* (%)				0.005
No	701 (67.27)	444 (70.59)	257 (62.23)	
Yes	341 (32.73)	185 (29.41)	156 (37.77)	
Chronic kidney disease, *n* (%)				<0.001
No	780 (74.86)	541 (86.01)	239 (57.87)	
Yes	262 (25.14)	88 (13.99)	174 (42.13)	
Chronic pulmonary disease, *n* (%)				<0.001
No	821 (78.79)	520 (82.67)	301 (72.88)	
Yes	221 (21.21)	109 (17.33)	112 (27.12)	
Congestive heart failure, *n* (%)				<0.001
No	732 (70.25)	508 (80.76)	224 (54.24)	
Yes	310 (29.75)	121 (19.24)	189 (45.76)	
GCS, *n* (%)				0.017
13–15	708 (67.95)	435 (69.16)	273 (66.10)	
3–8	132 (12.67)	65 (10.33)	67 (16.22)	
9–12	202 (19.39)	129 (20.51)	73 (17.68)	
Ventilation status, *n* (%)				0.292
No	366 (43.68)	218 (45.23)	148 (41.57)	
Yes	472 (56.32)	264 (54.77)	208 (58.43)	
30-day mortality, *n* (%)				<0.001
No	932 (89.44)	587 (93.32)	345 (83.54)	
Yes	110 (10.56)	42 (6.68)	68 (16.46)	
Length of stay (LOS)	6.99 (3.75, 12.83)	6.80 (3.54, 12.38)	7.71 (4.40, 13.70)	0.021

### Feature selection results

**Figure 2:**
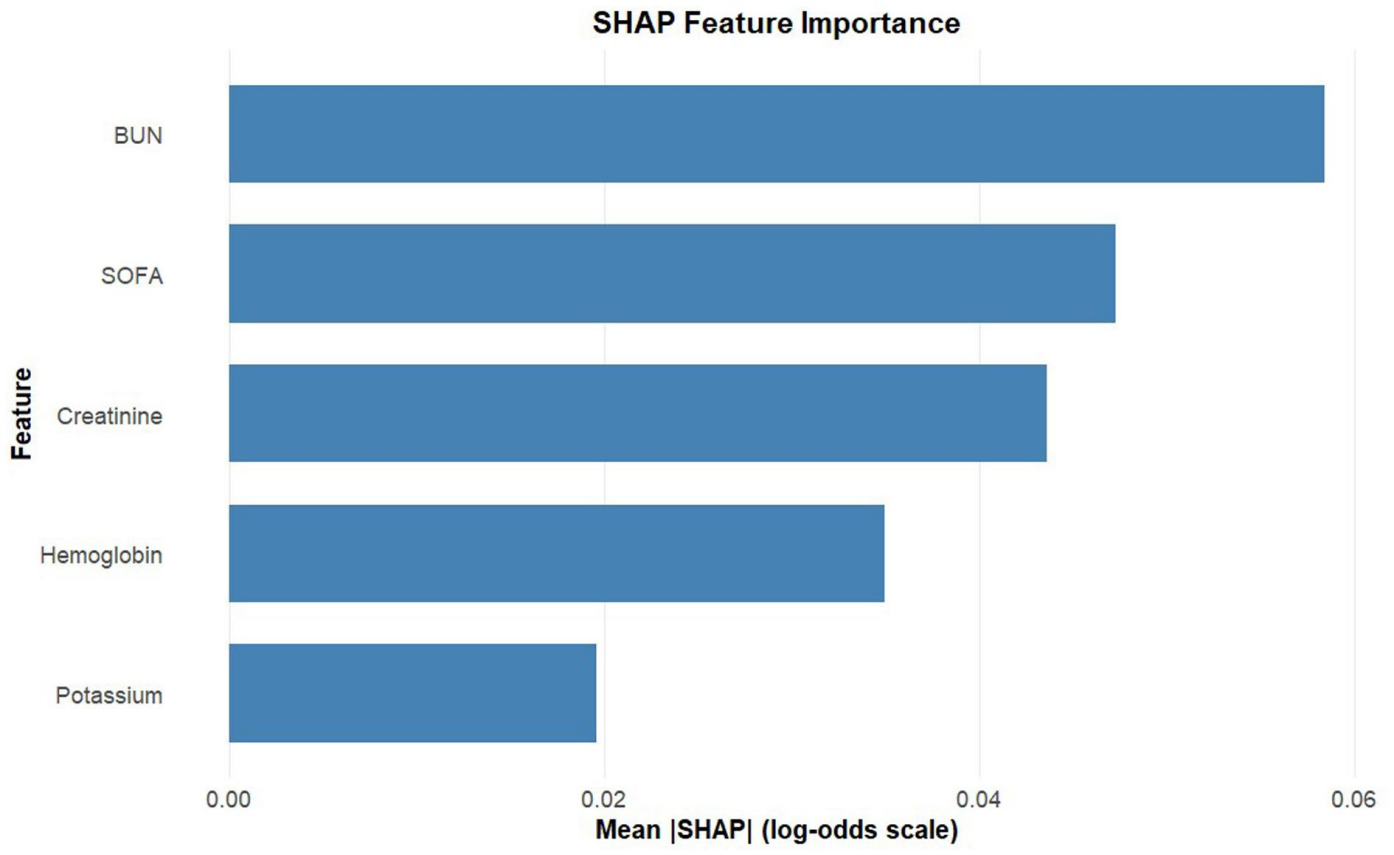
SHAP value distribution for each predictor. Positive SHAP values indicate increased odds of SMI while negative values indicate decreased odds. Creatinine and blood urea nitrogen (BUN) show strong positive associations with risk, and hemoglobin exhibits a protective effect. Trend lines reflect monotonic relationships, supporting the logistic model’s linearity assumptions.

**Figure 3:**
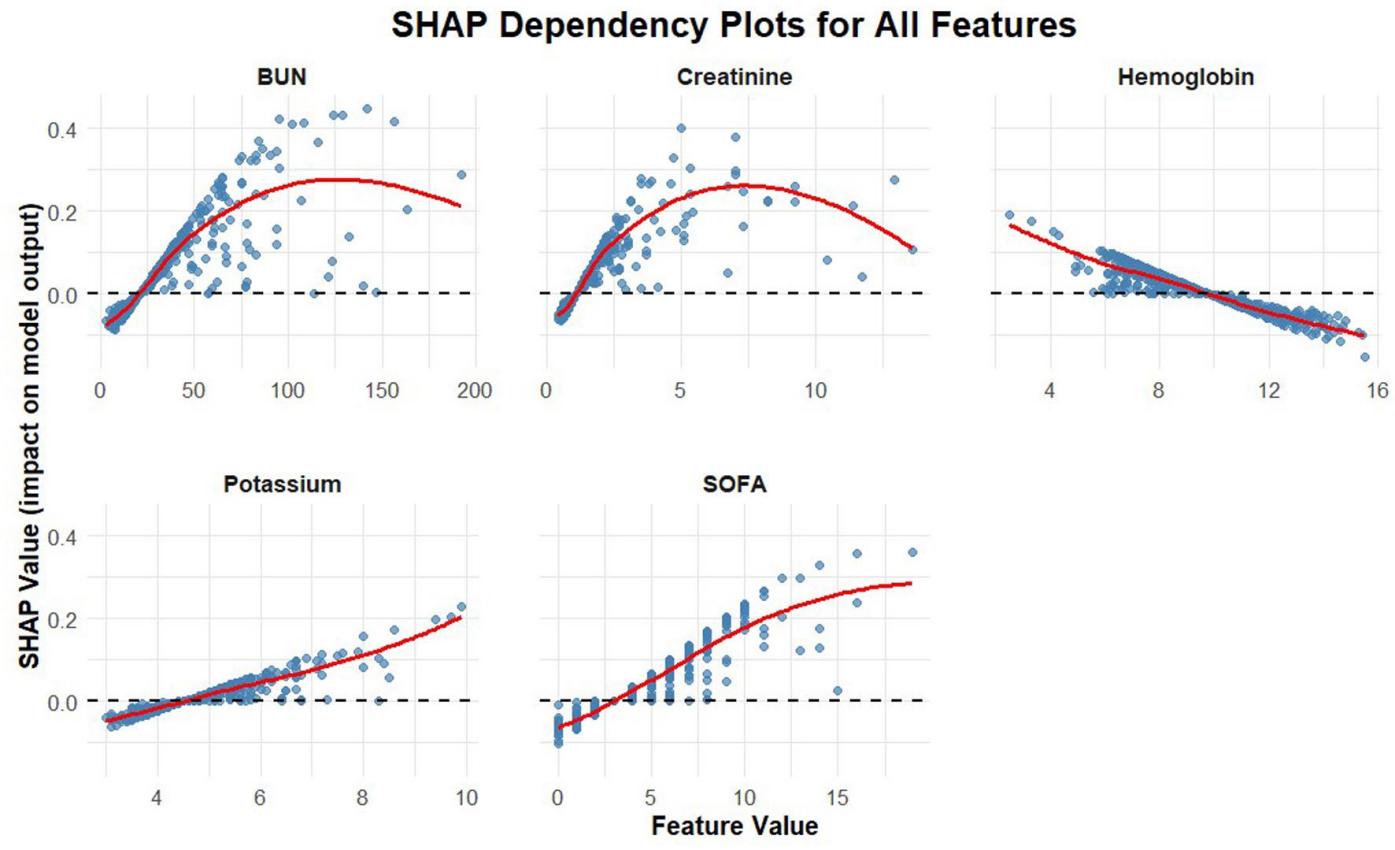
Mean absolute SHAP values of predictors. The ranking reflects the relative importance of each variable in predicting SMI, with BUN as the most influential factor, followed by SOFA score and creatinine.

Table 3 presents the results of the univariate logistic regression analysis and potential risk factors for myocardial injury identified through multivariate logistic regression modeling in the training set. The Multivariate logistic regression analysis identified several independent risk factors for myocardial injury, including age, history of kidney disease, SOFA score, serum creatinine, potassium, BUN, and hemoglobin (all *p* < 0.05). However, to balance statistical accuracy with clinical utility, we performed a secondary screening based on the principle of parsimony. While statistically significant, age is a non-modifiable factor. For renal function, creatinine provides a more objective, real-time assessment than history of kidney disease, avoiding data redundancy. Moreover, including these two variables increased the AUC by a negligible 0.011 (0.772 vs. 0.783). Therefore, to balance model performance and simplicity, “age” and “kidney disease” were excluded. The final nomogram comprises five clinically actionable predictors: BUN, hemoglobin, SOFA score, serum potassium, and creatinine. While the multivariate logistic regression model identified independent predictors and provided basic interpretability through Odds Ratios (ORs), it could not fully quantify the relative contribution of each variable or visually demonstrate the risk trends associated with continuous variable changes. Therefore, we utilized SHAP analysis, grounded in game theory, to decompose the model’s predictions into individual variable contributions. Figure 2 illustrates how individual variables affect the prediction for SMI injury. Higher levels of BUN, creatinine and SOFA score were associated with an increased risk, while higher hemoglobin levels suggested a protective effect. Figure 3 ranks the variables by their overall importance based on mean absolute SHAP values. BUN was the most significant predictor, followed by SOFA scores and creatinine, highlighting the critical role of renal function and systemic severity in predicting myocardial injury. These analyses provide additional insights into predictive models and help clarify the impact of key clinical parameters.

**Table 3:** Univariate and multivariate logistic regression analyses in the training set.

	Univariate analysis			Multivariate analysis		
Characteristics	OR	95% CI	*P* value	OR	95% CI	*P* value
Gender						
Female		Ref.				
Male	1.182	0.976–1.608	0.285			
Age	1.022	1.012–1.032	<0.001	1.029	1.015–1.043	<0.001
Invasive ventilation	1.066	0.779–1.459	0.688			
GCS						
13–15		Ref.				
9–12	0.941	0.638–1.390	0.761			
3–8	1.492	0.957–2.324	0.077			
Diabetes	1.340	0.976–1.841	0.071			
Chronic kidney disease	4.632	3.238–6.627	<0.001	1.783	1.049–3.030	0.033
Chronic pulmonary disease	1.913	1.330–2.751	<0.001	1.514	0.944–2.427	0.085
SOFA	1.246	1.169–1.328	<0.001	1.138	1.054–1.228	<0.001
DBP(mmHg)	0.985	0.972–0.999	0.032	1.003	0.984–1.021	0.785
SBP (mmHg)	0.995	0.985–1.004	0.270			
Heart rate	1.010	1.001–1.020	0.038	1.011	0.997–1.025	0.118
Resp rate	1.068	1.022–1.116	0.003	0.994	0.936–1.056	0.839
Temperature (°C)						
≥37.3		Ref.				
36.7–37.3	0.896	0.618–1.296	0.557	0.871	0.552–1.374	0.552
<36.7	1.635	1.073–2.490	0.022	1.438	0.845–2.466	0.181
Height (cm)	0.992	0.977–1.006	0.252			
Weight (kg)	0.999	0.994–1.005	0.806			
BMI (kg/m^2^)	0.991	0.967–1.016	0.489			
Hemoglobin (g/dL)	0.767	0.711–0.827	<0.001	0.877	0.799–0.962	0.006
WBC (K/μL)	1.026	1.005–1.048	0.015	1.020	0.993–1.047	0.144
Platelet (K/μL)	0.996	0.994–0.998	<0.001	0.999	0.996–1.001	0.308
RBC (m/μL)	0.520	0.420–0.645	<0.001	0.963	0.514–1.717	0.899
Lymphocytes (%)	1.027	0.967–1.090	0.387			
Bicarbonate(mEq/L)	1.037	0.997–1.079	0.074			
Creatinine (mg/dL)	3.760	2.761–5.119	<0.001	1.771	1.197–2.619	0.004
Aniongap (mEq/L)	1.068	1.032–1.105	<0.001	0.969	0.921–1.021	0.240
BUN (mg/dL)	1.053	1.041–1.065	<0.001	1.016	1.001–1.031	0.042
Calcium(mg/dL)	1.273	1.043–1.555	0.018	1.005	0.757–1.334	0.971
Glucose (mg/dL)	1.003	1.002–1.005	<0.001	1.001	0.999–1.003	0.193
Potassium (mEq/L)	1.672	1.394–2.006	<0.001	1.232	1.001to 1.516	0.049
Sodium (mEq/L)	1.024	0.994–1.055	0.115			
PT (s)	1.037	1.017–1.058	<0.001	1.000	0.981–1.019	0.965
PTT (s)	1.011	1.004–1.018	0.003	0.994	0.985–1.004	0.241
AST (IU/L)	1.002	1.001–1.004	0.003	0.999	0.994–1.003	0.849
ALT (IU/L)	1.003	1.001–1.006	0.010	1.005	0.997–1.010	0.280

IQR, interquartile range; GCS, Glasgow coma scale; SBP, systolic blood pressure; DBP, diastolic blood pressure; SOFA, sequential organ failure assessment; WBC, White blood cell; BUN, blood urea nitrogen; PT, prothrombin time; PTT, activated partial thromboplastin time; ALT, alanine aminotransferase; AST, aspartate aminotransferase.

### Construction and validation of the nomogram

**Figure 4:**
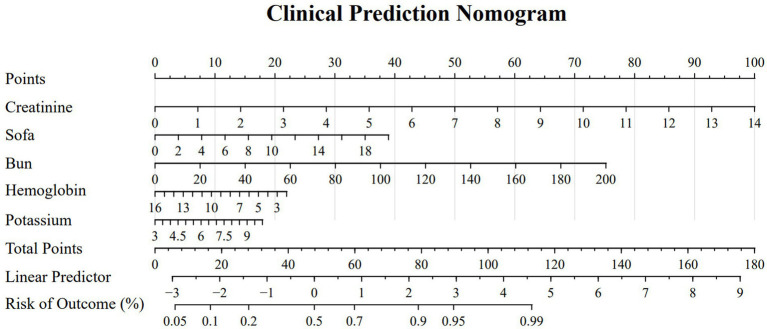
Nomogram for predicting the probability of myocardial injury in participants, where scores for urea nitrogen, hemoglobin, SOFA score, potassium, and creatinine level are assigned by drawing a line upward from their respective values to the “Points” line. The sum of all these scores plotted on the “Total Points” line corresponds to the predicted probability of myocardial injury occurrence in patients with traumatic brain injury.

**Figure 5:**
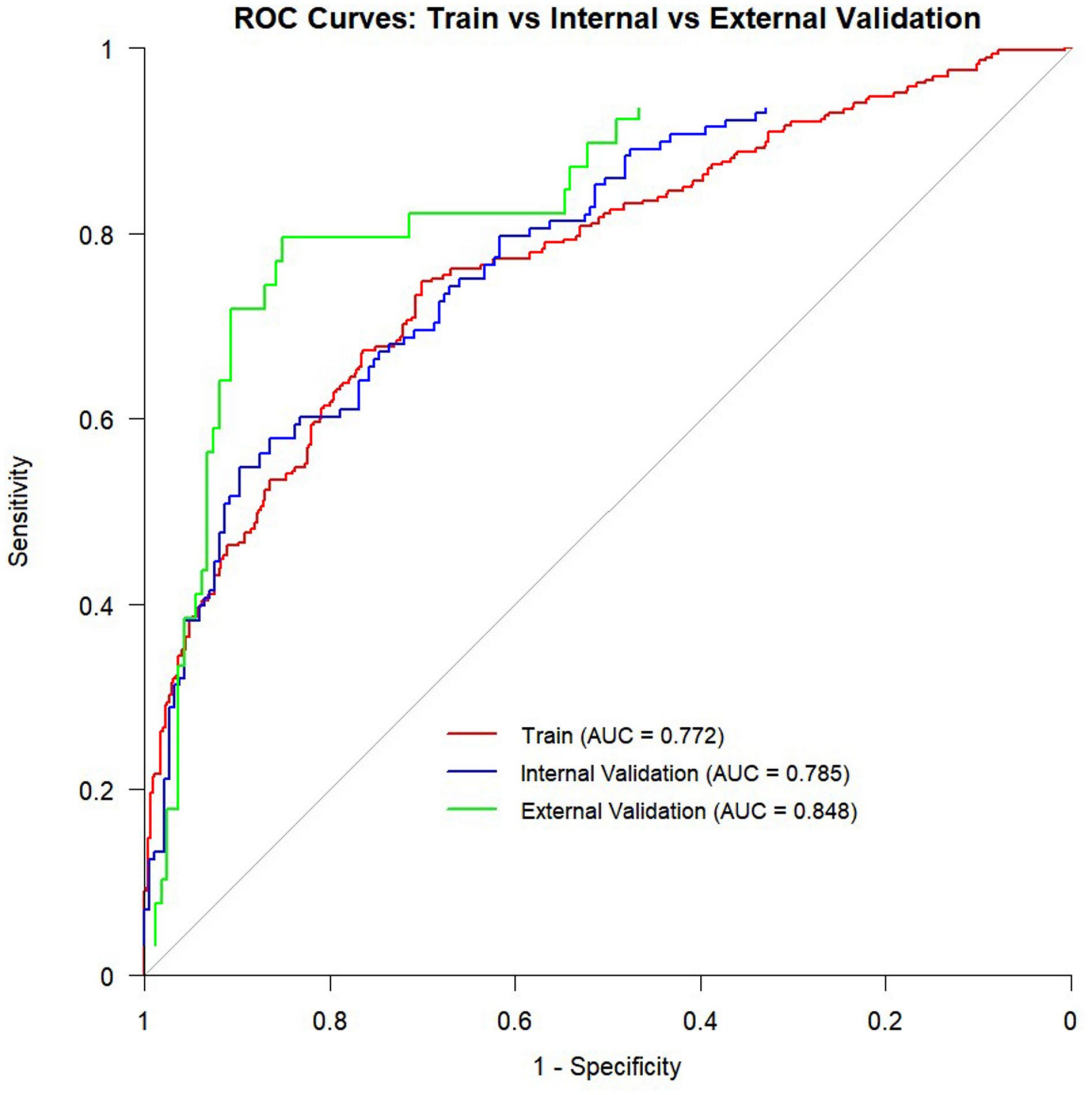
Receiver operating characteristic (ROC) curves for the training cohort, internal validation cohort, and external validation cohort. The AUC values were 0.772 (95% CI 0.737–0.808) for the training set, 0.785 (95% CI 0.733–0.837) for the internal validation set, and 0.848 (95% CI 0.778–0.917) for the external cohort.

**Figure 6:**
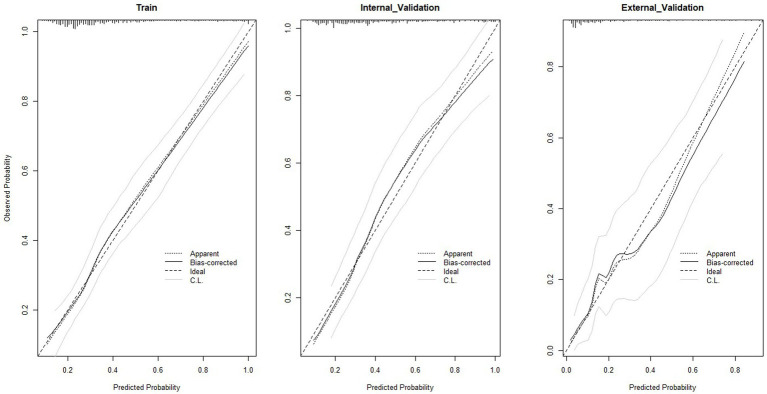
Calibration curves of the nomogram in the training set, internal validation set and external validation set. “Ideal” represents the ideal reference line of the nomogram, “Apparent” denotes its apparent predictive performance, and “Bias-corrected” refers to its predictive performance after bias correction.

**Figure 7:**
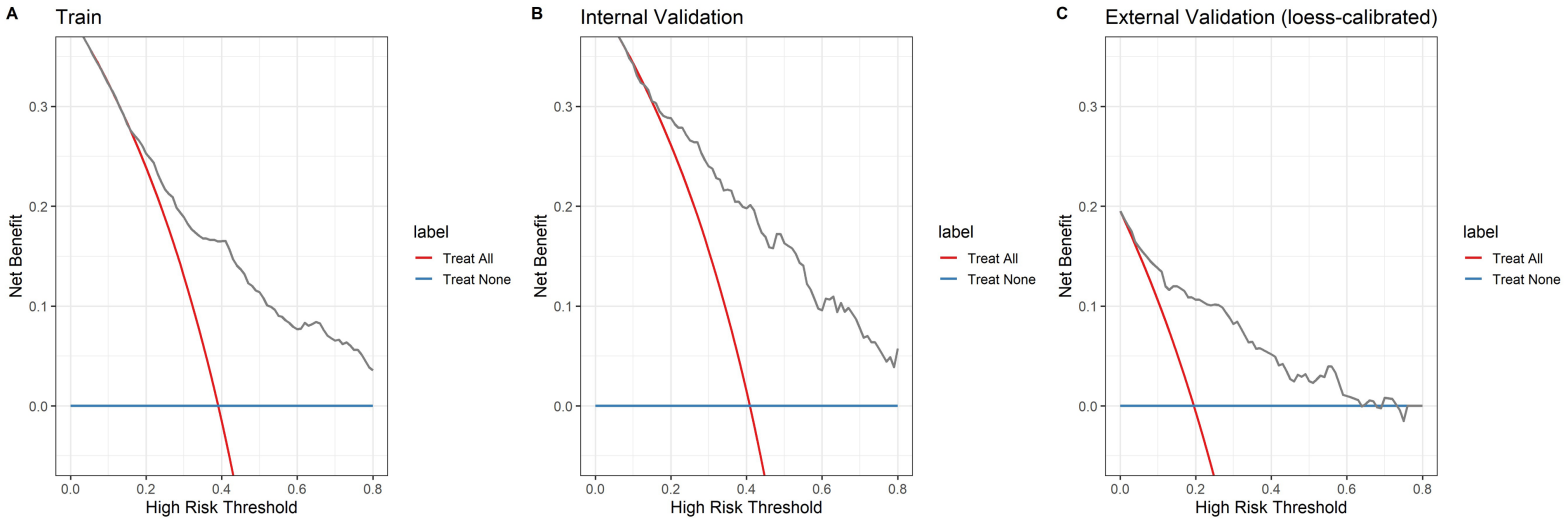
Decision curve analysis (DCA) illustrates the predictive performance of the model in the training set **(A)**, internal validation set **(B)**, and external validation set **(C)**. Here, the horizontal line represents the hypothetical scenario of “no participants experiencing myocardial injury,” while the gray diagonal line represents the scenario of “all participants experiencing myocardial injury.” The red and blue curves correspond to the nomogram’s prediction of myocardial injury risk. Across most probability thresholds, the decision curves based on this model (red and blue) demonstrate superior clinical net benefit compared to both the “intervene for all” and “intervene for none” strategies.

Based on the selected features, we constructed a logistic regression model and developed a nomogram (Figure 4). The total score is calculated by summing individual points assigned to each variable in the nomogram, with the corresponding probability indicating the risk of SMI. Model validation was performed using ROC curves, calibration curves, and decision curves. ROC curve analysis (Figure 5) showed AUCs of 0.772 (95% CI 0.737–0.808) in the training set, 0.785 (95% CI 0.733–0.837) in the internal-validation set, and 0.848 (95% CI 0.778–0.917) in the external cohort (200 patients, Zhejiang Provincial People’s Hospital, 2020–2025), confirming excellent separation between those who did and did not develop secondary myocardial injury. Calibration curves (Figure 6) plot predicted versus observed probabilities across risk deciles. Bias-corrected lines lie close to the 45° ideal in all three cohorts, and Hosmer-Lemeshow tests were non-significant (training *p* = 0.321; internal-validation *p* = 0.456; external-validation *p* = 0.387). Brier scores further supported best overall accuracy in the external set (0.1151 vs. 0.181 and 0.183 for training and internal validation, respectively). Decision-curve analysis (Figure 7) compares net benefit across threshold probabilities of 5–60%. The nomogram delivered higher net benefit than both “treat-all” and “treat-none” strategies in every cohort, with the largest advantage seen in the external validation, indicating that routine use of the model would reduce unnecessary interventions while still capturing patients at genuine risk of myocardial injury.

## Discussion

We analyzed clinical and survival data from 1,042 MIMIC-IV-derived TBI patients, identifying five independent predictors (SOFA score, hemoglobin, serum potassium, blood urea nitrogen, creatinine) via univariate and multivariate logistic regression to construct a nomogram for SMI prediction. Stratified analysis confirmed the clinical relevance of SMI, patients with SMI exhibited significantly worse systemic inflammation, multi-organ dysfunction, and disease severity, alongside higher 30-day mortality and prolonged hospitalization. These findings validate SMI as a critical prognostic indicator rather than a transient biomarker elevation. Our model demonstrates robust discrimination and calibration. Uniquely, it leverages routine data available within 24 h of ICU admission to quantify risk prior to detectable troponin elevation. This “pre-diagnostic” capability enables a shift from reactive assessment to proactive risk stratification, guiding early monitoring and targeted management of modifiable factors in high-risk patients.

Myocardial injury in TBI differs fundamentally from primary cardiac disease, with neurogenic stunned myocardium as the core mechanism—driven by excessive sympathetic activation and catecholamine storms post-TBI (3). Mechanistically, TBI mediates myocardial injury through distinct pathways. First, brain injury triggers inflammasome activation (e.g., NLRP3) in both the central nervous system and the heart. This activation promotes the release of cytokines like IL-1β via exosomes, subsequently inducing cardiac immune cell infiltration and inflammatory cascades. Splenectomy has been shown to alleviate this inflammatory response, thereby confirming the key role of the immune mechanism (22, 23). Second, autonomic dysfunction precipitates catecholamine surges and hemodynamic instability. Concurrently, increased intracranial pressure contributes to myocardial hypoxia. Together, these factors exacerbate cardiomyocyte apoptosis and electrophysiological abnormalities (4, 24).

Predicting myocardial injury in traumatic brain injury (TBI) patients faces three core challenges: delayed onset (requiring dynamic monitoring), ambiguous interpretation of traditional cardiac biomarkers in the TBI context, and confounding effects of TBI severity, systemic inflammation, and multi-organ dysfunction (11). These TBI-specific hurdles limit traditional cardiac risk tools, emphasizing the need for a tailored model—with our five identified predictors aligning closely with these mechanisms and challenges.

Anemia is common in traumatic brain injury (TBI) patients, especially within 48 h post-injury, and is strongly linked to higher mortality. This is primarily due to hemodilution from intravenous fluid resuscitation and extracranial bleeding (preoperatively and perioperatively). Notably, multiple studies confirm that anemia exacerbates myocardial injury (25, 26). Hemoglobin < 9 g/dL precipitates myocardial hypoxia, amplifies catecholamine storm, and increases risks of cardiac overload and myocardial hypertrophy (27, 28). Anemia is both a comorbidity and an important precipitating factor for myocardial injury. Anemia correction significantly improves long-term outcomes in TBI patients, reducing mortality and the incidence of cardiovascular complications (arrhythmias: 96% reduction, myocardial infarction: 81% reduction, heart failure: 86% reduction). Additionally, it reduces infectious complications such as pneumonia and urinary tract infections (29–31).

In addition, this study identified elevated blood urea nitrogen and creatinine levels as independent risk factors for SMI in TBI patients. Post-TBI, excessive sympathetic activation, enhanced systemic inflammation, and hemodynamic instability can rapidly impair renal function, manifested as increased BUN and creatinine levels (32, 33). The accumulation of these metabolites directly damages cardiomyocytes, promoting fibrosis and apoptosis via the NF-κB/NLRP3 inflammatory pathway and oxidative stress. Notably, experimental studies suggest that targeting this pathway (e.g., using rosuvastatin) can attenuate LPS-induced oxidative stress and reduce cardiac injury, highlighting the relevance of these metabolic markers (34–36). Clinical data confirm the prognostic value of these markers: patients with creatinine >1.3 mg/dL have a significantly higher 1-year mortality (25.9% vs. 6.8%, *p* = 0.002), and a > 30% increase in creatinine is linked to elevated risks of myocardial infarction, heart failure, and all-cause mortality (37, 38). Elevated BUN levels also have significant predictive value; for every unit increase in BUN level, the risk of death increases by 1.02-fold; higher BUN levels further increase the risk of death in heart failure patients by 2.29-fold (39). In acute myocardial infarction (AMI) complicated by cardiogenic shock (CS), elevated BUN is associated with short-term mortality and major adverse cardiovascular events (MACE) (40). Additionally, an increased BUN/Cr ratio independently predicts all-cause mortality (HR = 2.1) and is significantly associated with a decrease in LVEF (41).

Although potassium is linked to renal function, it independently regulates myocardial electrical activity and is critical in TBI-related cardiac injury. Hyperkalemia induces cardiomyocyte membrane depolarization, disrupts calcium balance, impairs myocardial contractility, and raises arrhythmia risk. Existing evidence supports a strong association between potassium levels and myocardial injury: hyperkalemia correlates with elevated hs-TnT in TBI patients (42). In rat models, potassium levels ≥6.0 mEq/L induced a 2.1-fold increase in cTnI (43). Among patients with acute coronary syndrome (ACS), those with potassium >5.0 mEq/L exhibited a 67% higher TnI positivity rate (OR = 1.67, 95% CI: 1.12–2.49) (44). And in heart failure patients, each 1 mEq/L increase in potassium corresponded to a 0.32 ng/mL elevation in TnT (*β* = 0.32, *p* = 0.003) (45). Furthermore, hyperkalemia has been extensively integrated into cardiovascular disease risk stratification, with its incidence positively correlating with cardiac function deterioration (46, 47). Our data further confirm this relationship: TBI patients with hyperkalemia (>5.0 mEq/L) demonstrated a myocardial injury incidence of 66%, significantly exceeding the 32% observed in the normokalemic group. Together with previous research, these findings confirm potassium as a valuable cross-disease marker for predicting myocardial injury.

The SOFA score, included as a core indicator of multi-organ dysfunction, is widely used to assess organ impairment in ICU patients. In recent years, it has demonstrated significant prognostic value in acute myocardial infarction, heart failure, and sepsis-related myocardial injury (48–50). This study confirmed the SOFA score as an independent predictor of post-TBI myocardial injury, consistent with existing clinical evidence and validating the close link between post-TBI myocardial injury, systemic inflammatory response, and multi-organ dysfunction. An elevated SOFA score essentially reflects the intensity of TBI-induced systemic inflammation—the key mediator of brain-heart axis-mediated myocardial injury—highlighting the model’s targeting of TBI-specific pathophysiological processes.

Compared with existing predictive models, our nomogram offers distinct innovations and advantages: First, it is the first model specifically developed for TBI patients, filling the gap in predictive tools for this cohort. It integrates indicators reflecting TBI-specific pathophysiology, such as the SOFA score (multi-organ dysfunction) and electrolytes (common post-TBI metabolic disturbances). Second, it balances good predictive performance (training set AUC = 0.772, validation set AUC = 0.796; favorable calibration and decision curves) with strong clinical applicability—relying solely on five routine clinical indicators obtainable within 24 h of ICU admission, no special tests required. Importantly, its “pre-diagnostic” feature overcomes the limitation of traditional biomarkers, enabling risk quantification before troponin elevation and shifting from “passive diagnosis” to “active stratification.”

Rather than defining a rigid protocol, our model serves as a hypothesis-generating tool for early risk stratification, potentially facilitating a shift from reactive diagnosis to proactive surveillance. Assessment can be completed shortly after ICU admission; patients with high-risk could be prioritized for serial hsTnT monitoring (to mitigate the risk of missed diagnoses from single-point testing) and individualized interventions for modifiable risk factors—correcting anemia to improve myocardial oxygenation, managing hyperkalemia to prevent arrhythmias, optimizing fluid management in renal dysfunction to reduce toxin accumulation, and early inflammation control in high-SOFA patients to block brain-heart axis injury pathways.

This study has limitations. First, the GCS score (TBI severity marker) was not statistically significant in multivariate analysis (*p* = 0.761, Table 3), and specific neuroimaging markers or intracranial pressure (ICP) data were unavailable or excluded due to high missing rates (>25%). Consequently, our nomogram is driven primarily by indicators of systemic illness and renal dysfunction (SOFA, Creatinine, BUN) rather than the anatomical brain injury phenotype itself. While the SOFA score implicitly captures hemodynamic instability (e.g., vasopressors), this observation suggests that SMI is fundamentally mediated by systemic “Brain-Heart-Kidney” crosstalk and generalized physiological stress, rather than solely by primary intracranial injury severity. Future studies integrating TBI-specific scores (e.g., IMPACT-Core) are needed to refine this neuro-cardiac association. Second, our definition of SMI relied solely on biochemical evidence (cardiac troponin elevation) without mandatory confirmation via echocardiography or electrocardiography (ECG) due to data limitations. While this aligns with the universal definition of “myocardial injury,” this approach may underestimate functional cardiac abnormalities, the nomogram is limited to early risk stratification and cannot replace cardiac enzyme testing—clinical application requires complementary use of both. Finally, inherent heterogeneity exists between the MIMIC-IV database and the external validation cohort, including differences in ICU practice patterns, specific troponin assays, and patient demographics. While we standardized the outcome definition using the 99th percentile rule, we could not perform a granular comparison of all baseline injury characteristics or management protocols due to data limitations. However, the model’s robust performance in the external cohort (AUC 0.848) suggests good generalizability despite these variations. Future research should focus on optimizing performance by integrating imaging indicators (e.g., echocardiographic strain rate) and novel biomarkers, as well as conducting prospective studies to validate the impact of early targeted interventions on patient outcomes.

## Conclusion

In conclusion, this study has successfully developed and validated a novel nomogram incorporating five clinical parameters (blood urea nitrogen, hemoglobin, SOFA score, serum potassium, and creatinine) for predicting SMI in TBI patients requiring ICU admission. This model, built on easily accessible initial clinical data, offers a potential tool for early risk stratification *prior to* the confirmation of myocardial injury by cardiac biomarkers. Its simplicity makes it a candidate for future integration into clinical workflows to guide monitoring intensity and preventive care. While external validation in a single-center cohort confirms its robustness, multicenter verification and prospective studies are needed to further support its clinical translation. Importantly, the nomogram is intended to complement, not replace, cardiac enzyme testing for definitive diagnosis.

## Data Availability

The original contributions presented in the study are included in the article/Supplementary material, further inquiries can be directed to the corresponding author.

## Footnotes

1
https://physionet.org/content/mimiciv/2.2/
